# Immediate Post-Procedural and Discharge Assessment of Mitral Valve Function Following Transcatheter Edge-to-Edge Mitral Valve Repair: Correlation and Association with Outcomes

**DOI:** 10.3390/jcm10225448

**Published:** 2021-11-22

**Authors:** Doron Sudarsky, Fabio Kusniec, Liza Grosman-Rimon, Ala Lubovich, Wadia Kinany, Evgeni Hazanov, Michael Gelbstein, Edo Y. Birati, Shemy Carasso

**Affiliations:** 1 The Lidya and Carol Kittner, B. Padeh Medical Center, Lea and Benjamin Davidai Division of Cardiovascular Medicine and Surgery, Poriya 15208, Israel; fkusniec@poria.health.gov.il (F.K.); l.grosman.rimon@gmail.com (L.G.-R.); alubovich@poria.health.gov.il (A.L.); WKinany@poria.health.gov.il (W.K.); EHazanov@poria.health.gov.il (E.H.); MGelbstein@poria.health.gov.il (M.G.); EBirati@poria.health.gov.il (E.Y.B.); SCarasso@poria.health.gov.il (S.C.); 2 The Azrieli Faculty of Medicine in the Galilee, Bar-Ilan University, Safed 1311502, Israel

**Keywords:** transcatheter edge-to-edge mitral valve repair, residual mitral regurgitation, mitral valve pressure gradient

## Abstract

The correlation between residual mitral regurgitation (rMR) grade or mitral valve pressure gradient (MVPG), at transcatheter edge-to-edge mitral valve repair (TEEMr) completion and at discharge, is unknown. Furthermore, there is disagreement regarding rMR grade or MVPG from which prognosis diverts. We retrospectively studied 82 patients that underwent TEEMr. We tested the correlation between rMR or MVPG and evaluated their association, with outcomes. Moderate or less rMR (rMR ≤ 2) at TEEMr completion was associated with improved survival, whereas mild or less rMR (rMR ≤ 1) was not. Patients with rMR ≤ 1 at discharge demonstrated a longer time of survival, of first heart failure hospitalization and of both. The correlation for both rMR grade (*r* = 0.5, *p* < 0.001) and MVPG (*r* = 0.51, *p* < 0.001), between TEEMr completion and discharge, was moderate. MR ≤ 2 at TMEER completion was the strongest predictor for survival (HR 0.08, *p* < 0.001) whereas rMR ≤ 1 at discharge was independently associated with a lower risk of the combined endpoint (HR 4.17, *p* = 0.012). MVPG was not associated with adverse events. We conclude that the assessments for rMR grade and MVPG, at the completion of TEEMr and at discharge, should be distinctly reported. Improved outcome is expected with rMR ≤ 2 at TEEMr completion and rMR ≤ 1 at discharge. Higher MVPG is not associated with unfavorable outcomes.

## 1. Introduction

Transcatheter edge-to-edge mitral valve repair (TEEMr) is a minimally invasive treatment for patients, suffering from symptomatic moderate-to-severe or higher mitral regurgitation (MR), who are deferred from surgery owing to either high or prohibitive surgical risk. Patients who are treated with TEEMr report improved functional capacity, exhibit reduced heart failure symptoms, and possibly have improved clinical course with less heart failure admissions and reduced mortality [1,2,3]. Efforts are being made to define the parameters that are associated with a significant clinical benefit to TEEMr treated patients. Encountering residual mitral regurgitation (rMR) or increase in the trans-mitral valve pressure gradient (MVPG), following TEEMr, is common and is associated with unfavorable clinical outcomes [3,4,5,6,7,8,9,10,11]. While achieving the lowest possible levels of both is advocated, the levels from which the risk for an adverse clinical course is mitigated, has been under investigation, bearing conflicting reported results [3,4,5,6,7,8,9,10,11,12,13,14,15,16]. Additionally, rMR and MVPG are assessed by either transesophageal echocardiography (TEE) immediately at the completion of TEEMr, or by transthoracic echocardiography (TTE) at any time between completion of the procedure and discharge. However, the correlation between these assessments of mitral valve function has not been investigated yet.

The objectives of the present study were: (1) to examine the relationship between post TEEMr TEE and TTE assessments of rMR or MVPG and long-term clinical outcomes; and (2) to evaluate the correlation between rMR and MVPG assessments, following TEEMr, by TEE at the completion of the procedure and by TTE at discharge.

## 2. Materials and Methods

### 2.1. Study Population

A retrospective analysis was conducted among patients who underwent TEEMr at Poriya Medical Center (PMC) between March 2015 and November 2020. The study complied with the ethical guidelines of the Declaration of Helsinki, was approved by the Ethical Review Board at PMC and each patient provided written informed consent before the intervention. Data were recorded prospectively during the index hospitalization and follow-up visits. All patients were diagnosed with either moderate-severe MR (MR-3) or severe MR (MR-4) and remained symptomatic despite optimal medical therapy and cardiac resynchronization therapy, when appropriate. Patients were evaluated by a multidisciplinary heart team and were deferred from surgical intervention due to high or prohibitive surgical risk. For those patients who required a repeated procedure, we included only the first procedure in the analysis and the additional procedure was registered as an adverse event.

### 2.2. TEEMr Procedure

TEEMr was performed under general anesthesia, using fluoroscopic and 2-dimensional (2D) and 3-dimensional (3D) TEE guidance. All procedures were performed with the Mitraclip^®^ device (Abbott Vascular, Santa Clara, CA, USA) using the standard technique previously described [1]. After the procedure, patients were transferred to an intermediate care unit or, if necessary, to the intensive cardiac care unit.

### 2.3. Echocardiography Assessment

A board-certified non-invasive cardiologist performed and interpreted echocardiography images using commercially available ultrasound systems (GE Vivid E9, GE Medical Systems, Milwaukee, WI, USA; Philips Epiq7CVx, Philips Medical System, Andover, MA, USA; Siemens Acuson SC2000 PRIME, Siemens Medical Solutions, Malvern, PA, USA). The assessments were performed by 2D TTE and TEE at baseline, by 2D and real-time 3D TEE during TEEMr, after the last clip was placed while the patient was still under the influence of general anesthesia, as well as by 2D-TTE again at discharge and at follow-up visits. Left ventricle (LV) volume and LV ejection fraction (LVEF) were assessed using the biplane Simpson’s method. LV end-diastolic diameter (LVEDD), LV end-systolic diameter (LVESD) and left atrial (LA) diameter were measured on M-mode in the parasternal long axis view. Systolic pulmonary pressures (SPAP) were estimated from measuring the pressure gradient between the right ventricle (RV) and right atrium (RA) in systole and estimated RA pressure using inferior vena cava size and collapsibility [17]. MR etiology was classified as primary MR (PMR), secondary MR (SMR) or mixed MR according to the underlying pathology and valve morphology. MR was graded, prior to intervention, following the American Society of Echocardiography criteria, as mild MR (MR-1), moderate MR (MR-2), moderately-to-severe MR (MR-3) and severe MR (MR-4) [18]. Specific indices of MR severity included the effective regurgitant orifice area (EROA), assessed by the proximal iso-velocity surface area (PISA) method and regurgitant volume. Overall MR grade was determined by integrating multiple parameters, including the above aforementioned parameters as well as MR mechanisms, jet size and eccentricity, mitral filling pattern and pulmonary venous flow pattern. After the procedure rMR was assessed according to the technique described by Foster et al. [19]. Trans-mitral gradient was measured by a continuous-wave (CW) Doppler of mitral inflow in diastole with the beam of the CW Doppler located at the center of the largest neo-orifice created after clipping. The average of 3 consecutive beats (or 5 consecutive beats if the patient had atrial fibrillation) was reported. MVPG was rounded in 0.5-mmHg increments in cases where the patient had atrial fibrillation during the examination and in cases where there were challenges in assessing MVPG owing to variations in angulation, acquisition and planimetry of the spectral Doppler envelope. Intraprocedural 3D TEE measurements were acquired from 3D TEE full-volume, color flow Doppler data sets.

### 2.4. Follow-Up and Outcomes

Technical success was assessed at exit from the catheterization laboratory while device and procedural success were measured after 30 days. All were defined according to the Mitral Valve Academic Consortium (MVARC) criteria [20]. Patients were followed in the outpatient clinic at 3 months, 6 months, and 12 months after TEEMr, and annually thereafter. The follow-up included an interview, vital signs measurements, physical examination, review of medications and TTE. Patients who were not able to attend follow-up at the clinic were interviewed over the telephone. New York Heart Association functional class (NYHA FC) was assessed at baseline and at all follow-up visits. Heart failure hospitalizations and survival status following the procedure were recorded.

### 2.5. Statistical Analysis

Data were analyzed using SPSS software, Version 18.0 (SPSS Inc., Chicago, IL, USA). The Shapiro–Wilk test was used to test the normality of distribution. The hypothesis of normality was rejected when the *p*-value ≤ 0.05. Continuous data with normal distribution were reported as the mean and standard deviation (SD) and were compared using either the dependent or independent samples *t*-test. Continuous variables with a non-normal distribution were reported as medians and interquartile ranges (IQR) and were compared using the Mann–Whitney U test for independent samples or the Wilcoxon signed-rank test for repeated measures. Categorical variables were reported as absolute numbers and percentages and were compared using the Chi-Square test for independent samples. McNemar’s Chi-square test was used to compare paired proportions. The correlation between TEE and TTE measurements of rMR grade and MVPG was assessed by Spearman’s rank correlation coefficients. Events-free survival was estimated using the Kaplan–Meier method and compared by the log-rank test. Univariable and multivariable Cox proportional hazards regression analyses were performed to evaluate for predictors of mortality and a combined endpoint of time to mortality, or first heart failure hospitalization with reported hazard ratios (HR) and 95% confidence intervals (CI). For multivariable analyses, an optimized model was calculated according to the results of the univariable analyses. Risk variables that were determined to be significant in the univariable analyses were tested subsequently with the multivariable modeling. A 2-sided *p*-value of ≤0.05 was considered statistically significant.

## 3. Results

### 3.1. Patient’s Characteristics

Eighty-two patients had undergone TEEMr at PMC between the years 2015 and 2020 and were included in the analysis. Baseline clinical characteristics are summarized in Table 1. The patients were stratified according to a cut-off of rMR-2 at the completion of TEEMr and according to a cut-off of rMR-1 at discharge.

Most patients (87.8%) were elderly (age > 65 years), and they suffered from multiple co-morbidities. All patients exhibited poor functional capacity and 43% had multiple heart failure related hospital admissions during the preceding year. Eight patients suffered from myocardial infarction complicated by severe mitral regurgitation unresponsive to PCI and medical therapy and they underwent urgent TEEMr during their index hospitalization.

Pre-procedural echocardiography parameters are presented in Table 2.

Fifty one (62.2%) patients had left ventricle (LV) systolic dysfunction and 19 patients (23.2%) had left ventricle ejection fraction (LVEF) of 30% or less. PMR was the underlying pathology in 23% of the patients with MR-4 and 5% of the patients with MR-3.

### 3.2. Procedural Course

One, two and three Mitraclip^®^ devices were deployed in 56 (68.3%), 22 (26.8%) and four (4.9%) patients respectively. Technical success was achieved in all cases. Seven patients underwent concomitant tricuspid valve repair. There were no intraprocedural deaths. One patient suffered from a large right to left shunt accompanied by hypotension and hypoxemia upon retraction of the delivery sheath from the left to the right atria. An atrial septal defect (ASD) occluder was immediately deployed with restoration of vital signs to normal. An ASD occluder was also used in another case to secure a large mobile mass that appeared after removal of the delivery system and was attached to the left side of the atrial septum. In another case, a patient suffered from sudden severe hypotension shortly after deployment of the device that necessitated cardio-pulmonary resuscitation with subsequent hemodynamic stabilization. TEE and fluoroscopy demonstrated a stable implanted device, rMR-1, low MVPG and no signs of cardiac tamponade. He was later discharged in a stable state. One patient suffered from a minor stroke that was recognized several minutes after extubation. Computed tomography did not demonstrate acute pathology. The patient was treated conservatively and over the course of several days, had resolution of all neurologic deficits.

### 3.3. Clinical Outcomes

**Figure 1:**
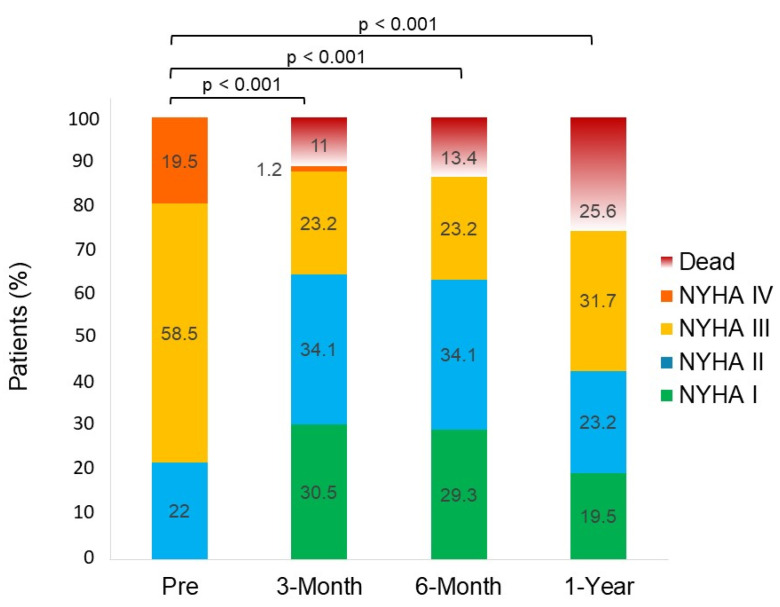
Changes in NYHA Functional class at baseline and at 3-Month, 6-Month and 1-Year after TEEMr.

Median follow-up was 433 (IQR 133–841) days. At 30 days, there were no cases of device embolization or single leaflet device attachment. Device success and procedural success were achieved in 66 (80.5%) and 63 (76.8%) of patients, respectively. Overall, four patients (4.9%) died within 30 days of TEEMr. One of them died after suffering from acute renal failure followed by dialysis and septic shock. Another one died from complications related to prolonged mechanical ventilation. The remaining two patients were discharged to their residencies and the cause of death was unknown. One patient who was discharged with aspirin and oral anticoagulant therapy, suffered from a hemorrhagic stroke 31 days following TEEMr, and later died 54 days after it. Another patient, that had procedural failure and ongoing heart failure symptoms, underwent surgical mitral valve replacement 67 days after the procedure. Three of the patients underwent repeated TEEMr at 47 days, 220 days and 323 days following their initial procedure. At the 1-year follow-up, 42.6% of surviving patients exhibited NYHA FC III and none of them had NYHA FC IV (Figure 1). While 68.3% of the patients had at least one heart failure admission during the preceding year, only 25% of them had heart failure related hospitalization after TEEMr (*p* < 0.001). A quarter of the patients died within one year, mostly from heart failure related causes. Overall, during the follow-up period, 23 patients died and 36 patients had either died or were hospitalized for worsening heart failure related symptoms.

### 3.4. MR and rMR Assessments

**Figure 2:**
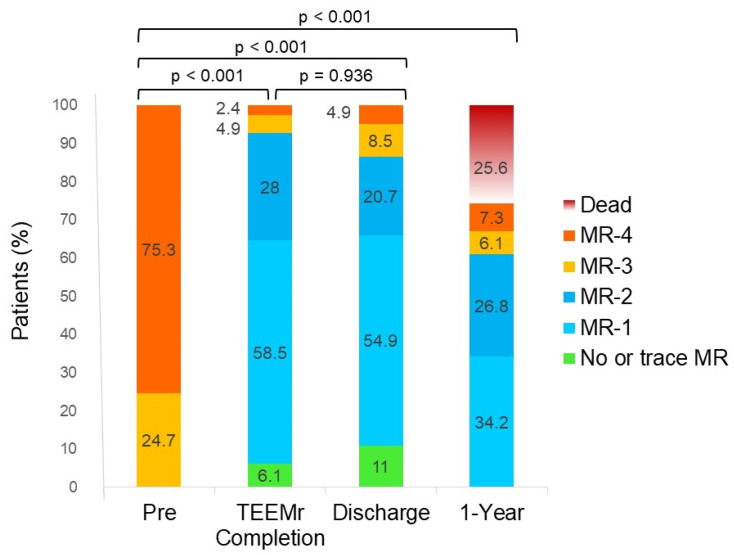
Mitral regurgitation grade at different time points. Columns illustrate the fractions of MR grades, of the cohort of patients, at baseline, at the completion of TEEMr, at discharge and at 1-year follow-up. rMR grades at completion of TEEMr, at discharge and at 1-year follow-up were significantly lower compared to pre-intervention MR grades. There was no significant difference in rMR grades at completion of TEEMr and at discharge. MR, mitral regurgitation; TEEMr, transcatheter edge-to-edge mitral valve repair.

MR and rMR grades prior to TEEMr, immediately after the completion of the procedure (by TEE) and the results of TTE assessments prior to discharge and at 1-year follow-up are presented in Figure 2.

At TEEMr completion, rMR ≤ 1 and rMR ≤ 2 was achieved in 64.6% and 92.7% of patients, respectively. The vast majority (90.5%) of the patients with baseline MR-4 and all the patients with baseline MR-3 had acute reduction of MR severity to rMR ≤ 2. More than half (55.6%) of the patients with baseline MR-4 and most (89.5%) of the patients with baseline MR-3 had acute reduction of MR severity to rMR ≤ 1. Compared to MR grades at baseline, rMR grades were significantly lower at completion of TEEMr (*p* < 0.001). At discharge, 65.9% of the patients had rMR ≤ 1 and 86.6% of the patients had rMR ≤ 2. rMR grades at discharge were also significantly lower compared to pre-procedure grades (*p* < 0.001). EROA and regurgitant volume at discharge were almost 50 percent lower compared to pre-TEEMr values (from 0.42 ± 0.23 cm^2^ to 0.22 ± 0.11 cm^2^ (*p* < 0.001) and from 58 ± 25.4 mL to 25.5 ± 24.6 mL (*p* < 0.001), respectively). There was no significant difference between rMR grades at completion of TEEMr and those at discharge (*p* = 0.936). Compared to rMR grade at the completion of TEEMr, rMR grade at discharge did not change in 57.3% patients and distributed almost equally between patients with higher (22%) or lower (20.7%) rMR grades. The correlation between rMR grades at completion of TEEMr and those at discharge was moderate (Spearman’s coefficient of rank correlation *r* = 0.5, *p* < 0.001). One year after TEEMr, most surviving patients (90.9%) retained lower levels of rMR grade compared to their pre-procedure grade (*p* < 0.001) Approximately two thirds of them (63.6%) had rMR ≤ 2 and about half of them had rMR ≤ 1.

### 3.5. MVPG Assessments

**Figure 3:**
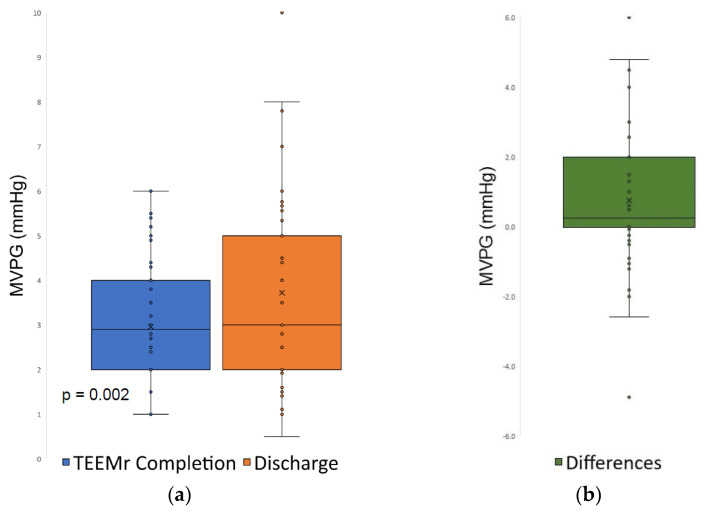
Mitral valve pressure gradient at completion of TEEMr and at discharge. (**a**) Changes in MVPG at TEEMr completion and at discharge. The cross-line marks the median of the measurements (50% quartile). The boxes represent the 25% to 75% interquartile values. The whiskers mark the smallest and largest measurements. The circles represent outliers. MVPG increased significantly (from 3 ± 1.4 mmHg at the completion of TEEMr to 3.7 ± 1.9 at discharge; *p* = 0.002); (**b**) Box plot of differences; MVPG, Mitral valve pressure gradient; TEEMr, Transcatheter edge-to-edge Mitral valve repair.

MVPG at discharge was higher compared to the MVPG at the completion of TEEMr (3.7 ± 1.9 mmHg vs. 3.0 ± 1.4 mmHg, *p* = 0.002) (Figure 3).

**Figure 4:**
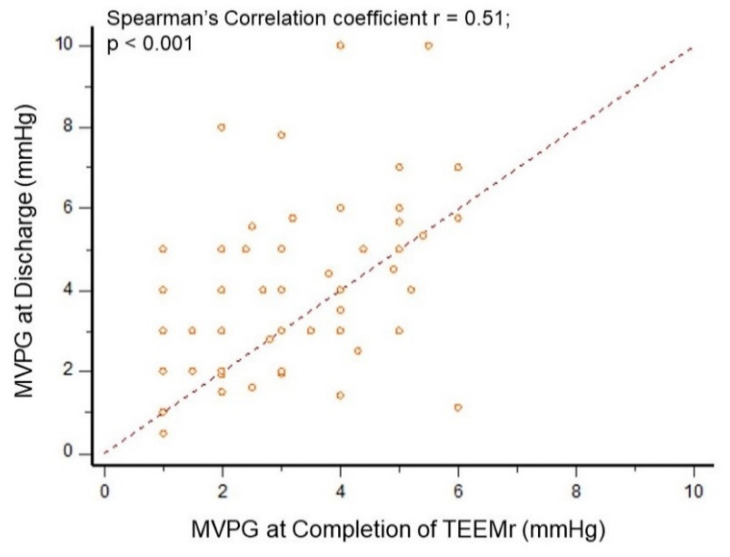
MVPG Correlation analysis at completion of TEEMr and at discharge. Scatter plots for Spearman’s correlation analysis between MVPG assessed by TEE at completion of TEEMr and MVPG assessed by TTE at discharge. Filled circles represent individual measurements. MVPG, Mitral valve pressure gradient; TEE, transesophageal echocardiography; TEEMr, Transcatheter edge-to-edge mitral valve repair; TTE, Transthoracic echocardiography.

Compared to MVPG at TEEMr completion, MVPG at discharge, did not change in about two-thirds (65.3%) of patients, was higher in 27.8% patients and was lower in 6.9% patients. The correlation for MVPG assessments was also moderate (Spearman’s coefficient of rank correlation *r* = 0.51, *p* < 0.001), (Figure 4).

### 3.6. Event Free Survival Analysis and Predictors of Outcomes

**Figure 5:**
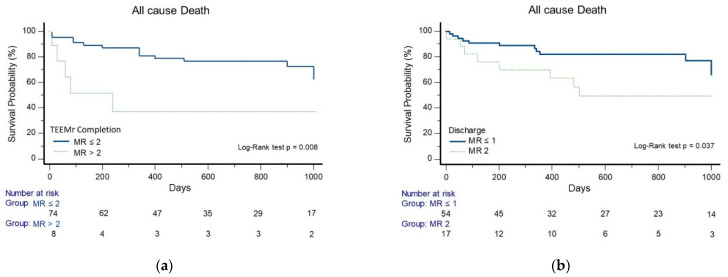

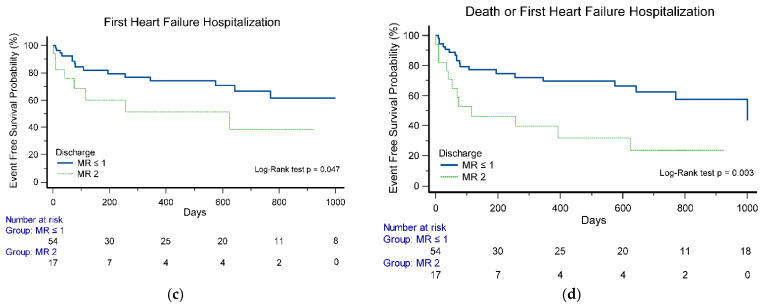
Kaplan-Meier analyses for clinical endpoints. (**a**) Survival probability stratified by Immediate post-procedural rMR grade; (**b**) Survival probability stratified by rMR grade at discharge; (**c**) Freedom from first heart failure related hospitalization stratified by rMR grade at discharge; (**d**) Freedom from death or first heart failure related hospitalization stratified by rMR grade at discharge.

Survival rates, by the Kaplan–Meier analysis, were higher in patients with rMR ≤ 2 at completion of TEEMr than those with rMR > 2 at completion of TEEMr (log-rank test, *p* = 0.008), (Figure 5a). There was no survival difference between patients with rMR ≤ 1 and patients with rMR-2, at completion of TEEMr (Log-rank test, *p* = 0.238), nor in the time to the first heart failure hospitalization (Log-rank test, *p* = 0.363) or in the time to the combined endpoint of all cause death, or first heart failure hospitalization (Log-rank test, *p* = 0.568). Patients with rMR ≤ 1 at discharge, compared to patients with rMR-2 at discharge, demonstrated longer time to all-cause death (Log-rank test, *p* = 0.037) (Figure 5b), time to the first heart failure hospitalization (Log-rank test, *p* = 0.047) (Figure 5c) and in the time to the combined end-point (Log-rank test, *p* = 0.003) (Figure 5d).

There were no differences, in any of the endpoints, between patients with MVPG ≤ 4.4 mmHg compared to patients with MVPG > 4.4 mmHg, or between patients with MVPG ≤ 5 mmHg compared to patients with MVPG > 5 mmHg, neither at completion of TEEMr nor at discharge. A multivariable cox proportional hazard regression model identified rMR ≤ 2 at TEEMr completion as the strongest independent predictor for survival with a 92% reduced risk of mortality (HR 0.08, 95% CI 0.02 to 0.28, *p* < 0.001) (Table 3(a)). rMR ≤ 1 at discharge, compared to rMR-2, was associated with more than a fourfold lower risk of the combined end point (HR 0.24, 95% CI 0.08–0.73, *p* = 0.012) (Table 3(b)).

## 4. Discussion

The main findings of this study are as follows. (1) rMR ≤ 2 at completion of TEEMr and rMR ≤ 1 at discharge were associated with improved mid-term clinical outcomes. (2) MVPG was not associated with an unfavorable outcome. (3) Following TEEMr, the correlation between either rMR grade or MVDG, assessed by TEE immediately at completion of the procedure and by TTE at discharge, is modest.

rMR, following TEEMr, has been strongly associated with poorer prognosis. However, the grade of rMR from which prognosis is diverted is still uncertain with conflicting reported results [1,2,3,4,5,6,7,12,13,14,15,16,21]. Some studies reported that a cutoff of rMR ≤ 2 was associated with improved clinical outcome. Kaneko et al. described that PMR patients and DMR patients with acute procedural success (APS), defined as rMR ≤ 2, had better survival than patients with acute procedural failure (rMR > 2) [4]. Capodano et al. found that achievement of APS was associated with a lower risk of mortality or a combined end-point that incorporated death and heart failure hospitalizations at a median follow-up of 12 months [15]. In a study by Lim et al., PMR patients with rMR-2 had a higher 12-month survival rate than patients with rMR > 2. Furthermore, in that study, patients with rMR-2 had similar survival rates to patients discharged with rMR ≤ 1 [12]. Similar results for a combined end-point have been described by Neuss et al., studying patients with either PMR or SMR [7]. In the Endovascular Valve Edge-to-Edge Repair (EVEREST) study, there were also no differences in patient outcomes between rMR-1 and rMR-2 [1]. However, in contrast, other studies found that patients with rMR-2 had less favourable outcomes compared to those with rMR ≤ 1. In these studies, rMR-2 was associated with long-term MR recurrence, less relief of symptoms, increased rate of heart failure related hospital admissions and ultimately worse survival [3,5,6,8,13,16]. Additionally, achieving optimal MR reduction, with TEEMr, is often counterbalanced against the risk of increasing the MVPG, compared to preprocedural values [7,8,22,23,24]. Higher MVPG has also been inconsistently associated with poorer long-term outcomes [7,8,9,10,22,23,24,25]. Neuss et al. reported that a combination of rMR-1 with a MVPG > 4.4 mmHg was associated with worse clinical outcomes compared to rMR-2 in combination with a MVPG ≤ 4.4 mmHg [7]. A recent trial found that Intraprocedural MVPG was actually clinically more influential than rMR > 2 [10]. Based on these trials, higher MVPG should be avoided even at the cost of achieving suboptimal reduction of MR. Conversely, a study by Patzelt et al. reported opposing results in which a less favorable clinical outcome was observed for the combination of rMR-2 and MVPG ≤ 4.4 mmHg, than for MR ≤ 1 and MVPG > 4.4 mmHg. While rMR was a predictor for a combined end-point in the entire cohort, MVPG proved predictive of clinical outcomes only in SMR patients [8]. In the EVEREST I pilot study and in the randomized EVEREST II study, following TEEMr, there was an increase in the MVPG, but this was not associated with clinically significant mitral stenosis [22,25]. Another recent publication reported that among SMR patients from the COAPT trial, following TEEMr, higher MVPG was not associated with increased risk of mortality, HF hospitalization or with the composite of both [9]. According to these reports, achieving ideal rMR reduction should be favored over the need to retain low MVPG. Notably, rMR or MVPG assessments, in most of these studies, were obtained by TTE performed anytime in the post procedural period between completion of TEEMr and discharge. The results of these studies directly influence intra-procedural real-time decisions such as moving a clip to a different position, adding a clip in the case of suboptimal MR reduction, or refraining from deployment of additional clips in the case of increased MVPG. At the base of these practices lies the assumption that after TEEMr, there is a close similarity between intraprocedural TEE and ex-post-facto TTE assessments of rMR grade and MVDG. However, according to our study, intra-procedural TEE assessments of rMR grade and MVPG correlate only modestly with those of post-procedural TTE assessments. There have only been a few reports which evaluated the concordance of MR severity by TTE and TEE [26,27,28]. Similarly, in these studies, the correlation between TTE and TEE assessments of MR grade was also modest, but none were conducted in the setting of TEEMr. Grayborn and colleagues found a moderate correlation between TEE and TTE for overall MR grade in patients with ischemic cardiomyopathy participating in the Surgical Treatment for Ischemic Heart Failure (STICH) trial (Spearman’s Correlation coefficient *r* = 0.51) [26]. The assessments of MR grade, in that trial, were made prior to intervention. Saiki et al. described that in patients who underwent surgical mitral valve reconstruction, intraoperative TEE measures of rMR severity correlated moderately with those obtained with post-operative TTE (Pearson’s Correlation coefficient *r* = 0.66) [28]. Potential contributors to the modest correlation, observed in our and in other reports, include different orientations of the imaging planes between TEE and TTE and timing of evaluation with a temporal delay between TEE and TTE. Furthermore, MR and MVPG are dynamic and vary with changes in parameters influencing the loading conditions such as heart rate, blood pressure, volume status, ventilation with increased expiratory pressure leading to elevated central venous pressure and the effect of anesthesia [26,29,30,31,32]. Additionally, established parameters, like EROA and regurgitant volume, which are part of the parameters used in the evaluation of the severity of native valve MR, may not be valid to evaluate rMR, following TEEMr, due to the complex neo-valve created with disrupted geometry, at least a double orifice and often two or more merging regurgitant jets [18,29,33]. The intergraded evaluation of rMR, following TEEMr, relies heavily on a semi-quantitative assessment. Therefore, it is susceptible to subjective interpretation and is a source for the variability of reports [18,29]. Likewise, MVPG is also deeply influenced by the changes in the loading conditions, between assessments, as well as by MR severity itself because the trans-mitral pressure gradient is a function of the square of the trans-valvular flow rate [31,32,34,35]. Decreased diastolic filling time during tachycardia, severe MR, high trans-mitral flow because of hyperdynamic states or anemia, for instance, will increase the MVPG. Furthermore, optimal alignment of the Doppler beam and flow, following the post-clipping deformation of the MV, where flow may be eccentric, is challenging and my affects MVPG measurement. We also found that pre-discharge MVPG, assessed TTE, increased compared to immediate post-intervention MVPG assessed by TEE. Similar results were described by Biaggi and colleagues even though MVPG, in that study, was not significantly influenced by an increase in heart rate from post intervention to the time of discharge or by a decrease in hemoglobin from pre-procedure to discharge values [24]. Large post TEEMr iatrogenic ASD with a shunt from the left atrium to right atrium is associated with immediate reduction in left atrial pressure [36]. Additionally, the prevalence and severity of post TEEMr ASD is reduced over time [37]. Both of these processes may also contribute to increased MVPG at discharge compared to MVPG at the completion of TEEMr.

### Study Limitations

Since this is a single-center, retrospective, observational study, it is possible that confounding factors have influenced the results. The study is of limited sample size and may be underpowered to accurately evaluate the clinical endpoints, thus affecting the results. A study with a larger sample size and higher statistical power may yield stronger correlations for either rMR grade or MVDG, assessed by both TEE immediately at completion of the procedure and by TTE at discharge. The clinical effect of elevated MVPG in a range compatible with severe mitral stenosis, following TEEMr, was not evaluated in this study. The regurgitant fraction was assessed only in cases where additional information was needed in order to determine MR level. Divergences in heart rate, blood pressure, hemoglobin level and the presence of post trans-septal atrial septal defect (ASD), with accompanying shunt, that may contribute to the differences between echocardiography assessments, were also not included in the present analysis. A more extensive study with a larger sample is needed in order to validate our results.

## 5. Conclusions

rMR grade, as well as MVPG, assessed by TEE at the completion of TEEMr and by TTE at discharge, correlate modestly and therefore should be distinctly reported. Future reports of these parameters are encouraged to include information regarding the timing and the modality of assessment. Achieving rMR ≤ 2, immediately at the completion of TEEMr may be a sufficient result, while encountering higher MVPG does not seem to be associated with an unfavorable outcome. These observations may aid with practical decisions taken in real-time during TEEMr. Additional larger studies are needed to test the association between rMR grade, MVPG or their combination, at TEEMr completion, and long-term outcomes. The association of rMR ≤ 1, by TTE, at discharge, with improved clinical course was further validated by this report.

## Figures and Tables

**Table 1:** Baseline clinical characteristics.

		TEEMr Completion (TEE)			Pre-Discharge (TTE)		
		rMR ≤ 2 (*n* = 74)	rMR > 2 (*n* = 8)	*p* Value	rMR ≤ 1 (*n* = 54)	rMR > 1 (*n* = 28)	*p* Value
Age (years)		75.2 ± 8.8	75.5 ± 7.5	0.921	75.2 ± 11.3	75.3 ± 9.3	0.982
Male		40 (54.1%)	6 (75%)	0.456	29 (53.7%)	17 (60.7%)	0.641
Body Mass Index (kg/m^2^)		27.8 ± 4.7	26.2 ± 4.4	0.39	28.2 ± 5.2	25.6 ± 4.6	0.119
Diabetes Mellitus		34 (45.9%)	3 (37.5%)	0.724	25 (46.3%)	13 (46.4%)	1
Hyperlipidemia		61 (82.4%)	7 (87.5%)	1	44 (81.5%)	24 (85.7%)	0.762
Hypertension		63 (85.1%)	5 (62.5%)	0.132	45 (83.3%)	23 (82.1%)	1
Smoking History		22 (29.7%)	2 (25%)	1	14 (25.9%)	10 (35.7%)	0.444
Ischemic Heart Disease		49 (66.2%)	5 (62.5%)	1	35 (64.8%)	19 (67.9%)	0.812
Past Myocardial Infraction		40 (54.1%)	5 (62.5%)	0.724	27 (50%)	18 (64.3%)	0.249
Past Coronary Intervention	PCI	37 (50%)	4 (50%)	1	25 (46.3%)	16 (57.1%)	0.485
	CABG	14 (18.9%)	3 (37.5%)	0.353	11 (20.4%)	6 (21.4%)	1
Past CVA or TIA		17 (23%)	2 (25%)	1	13 (24.1%)	6 (21.4%)	1
COPD		12 (16.2%)	3 (37.5%)	0.157	7 (13%)	8 (28.6%)	0.13
NYHA FC	II	17 (23%)	1 (12.5%)	0.117	15 (27.8%)	4 (14.3%)	0.353
	III	45 (60.8%)	3 (37.5%)		29 (53.7%)	19 (67.9%)	
	IV	14 (18.9%)	4 (50%)		10 (18.5%)	5 (17.9%)	
Past Year Heart Failure Admission (s)		53 (71.6%)	3 (37.5%)	0.102	38 (70.4%)	18 (64.3%)	0.622
Atrial Fibrillation		35 (47.3%)	4 (50%)	1	27 (50%)	12 (42.9%)	0.643
Pacemaker or Defibrillator		21 (28.4%)	2 (25%)	1	17 (31.5%)	6 (21.4%)	0.44
eGFR (mL/min/1.73 mr^2^) ^①^		56.1 ± 21.5	52.7 ± 22.7	0.69	55 ± 21.6	48.9 ± 23.6	0.247
Chronic Kidney Disease ^②^		44 (59.5%)	5 (62.5%)	0.719	29 (53.7%)	20 (71.4%)	0.156
Hemoglobin (g/dL)		11.3 ± 1.5	10.2 ± 1.8	0.06	11.4 ± 1.5	10.9 ± 1.7	0.211
Albumin (g/dL)		3.7 ± 0.5	3.5 ± 0.6	0.3	3.7 ± 0.5	3.6 ± 0.5	0.442
Medications	SAPT	23 (31.1%)	3 (37.5%)	0.704	15 (27.8%)	11 (39.3%)	0.324
	DAPT	17 (23%)	1 (12.5%)	0.678	12 (22.2%)	6 (21.4%)	1
	Oral Anticoagulant	36 (48.6%)	5 (62.5%)	0.712	28 (51.9%)	13 (46.4%)	0.816
	ACE-I/ARB/ARNI	44 (59.5%)	2 (25%)	0.13	37 (68.5%)	14 (50%)	0.149
	Beta Blockers	62 (83.8%)	7 (87.5%)	1	44 (81.5%)	25 (89.3%)	0.527
	Spironolactone	33 (44.6%)	3 (37.5%)	1	26 (48.1%)	10 (35.7%)	0.351
	Loop Diuretic	66 (89.2%)	7 (87.5%)	1	49 (90.7%)	24 (85.7%)	0.483
Urgency of Procedure ^③^	Urgent	9 (12.2%)	2 (25%)	0.199	7 (13%)	4 (14.3%)	0.469
	Expedited	2 (2.7%)	1 (12.5%)		1 (1.8%)	2 (7.1%)	
	Elective	63 (85.1%)	5 (62.5%)		46 (85.2%)	22 (78.6%)	
Cardiogenic Shock Within 30 Days		7 (9.5%)	2 (25%)	0.211	6 (11.1%)	3 (10.7%)	1
ACS Within 90 Days		11 (14.9%)	2 (25%)	0.607	8 (14.8%)	5 (17.9%)	0.756
PCI Within 30 Days		7 (9.5%)	2 (25%)	0.211	5 (9.3%)	4 (14.3%)	0.483
Surgical Risk	STS Score (%) ^④^	5.2 (2.8–9.0)	12.7 (4.1–17.6)	0.136	4.3 (2.6–9.2)	6.2 (3.8–11.4)	0.18
	EuroSCORE II (%)^⑤^	6.8 (4.0–12.4)	10.7 (6.1–13.8)	0.368	6.2 (3.2–11.9)	9.3 (5.6–13.5)	0.103

Baseline clinical data is stratified according to a cut-off of rMR-2 at the completion of TEEMr and according to a cut-off of rMR-1 at discharge. Continuous variables are presented as mean and standard deviation. Categorical variables are presented as percentages and absolute numbers. TEEMr, Transcatheter edge-to-edge mitral valve repair; TEE, Transesophageal echocardiography; TTE, Transthoracic echocardiography; PCI, Percutaneous coronary intervention; CABG, Coronary artery bypass graft surgery; CVA, cerebrovascular accident; TIA, transient ischemic attack; COPD, Chronic obstructive pulmonary disease; NYHA FC, New-York Heart Association functional class; eGFR, Estimated glomerular filtration rate; CKD, Chronic kidney disease; SAPT, Single antiplatelet therapy; DAPT, Dual antiplatelet therapy; ACE-I, angiotensin converting enzyme inhibitor; ARB, Angiotensin II receptor blocker; ARNI, angiotensin receptor-neprilysin inhibitor; ACS, Acute coronary syndrome; STS, Society of thoracic surgeons. ^①^ eGFR was estimated with the EPI CKD equation; ^②^ CKD was defined, in correspondence with the National Kidney Foundation eGFR stages for end stage renal disease, as eGFR < 60 mL/min/1.73 mr^2^; ^③^ Urgency of procedure was defined according to the National Confidential Enquiry into Patient Outcome and Death (NCEPOD) classification; ^④^ Society of Thoracic Surgeons (STS) scores for the risk of death within 30 days after mitral-valve repair; ^⑤^ EuroSCORE II scores for the risk of in-hospital mortality after cardiac surgery.

**Table 2:** Baseline echocardiography parameters taken prior to TEEMr.

		TEEMr Completion (TEE)			Pre-Discharge (TTE)		
		rMR ≤ 2 (*n* = 74)	rMR > 2 (*n* = 8)	*p* Value	rMR ≤ 1 (*n* = 54)	rMR > 1 (*n* = 28)	*p* Value
Left Ventricle End Diastolic Volume (mL)		135.9 ± 45.3	151.2 ± 38.8	0.434	130 ± 42.3	152.7 ± 45.7	0.046
Left Ventricle End Systolic Volume (mL)		79.8 ± 37.5	82.8 ± 23.8	0.847	77.3 ± 36.9	86 ± 34.2	0.347
Left Ventricle End Diastolic Diameter (mm)		59.6 ± 8.1	58 ± 7.9	0.622	59 ± 8.3	60.4 ± 7.7	0.448
Left Ventricle End Systolic Diameter (mm)		46.4 ± 10.2	44.7 ± 9.2	0.688	45.7 ± 10.2	47.1 ± 10.1	0.56
Left Ventricle Ejection Fraction (%)		44.3 ± 15.1	46.3 ± 9.2	0.725	44.2 ± 14.6	45.1 ± 14.7	0.786
Left Atrium Volume (mL)		98.3 ± 32.5	63.8 ± 19.1	0.014	94.9 ± 33.1	97 ± 32.7	0.794
Left Atrium Volume Index (mL/mr^2^)		54.8 ± 16.6	37.2 ± 13.4	0.015	52.3 ± 16.8	55.7 ± 17.3	0.437
Mitral Regurgitation Mechanism	Primary	23 (31.1%)	3 (37.5%)	0.873	17 (31.5%)	9 (28.6%)	0.385
	Secondary	44 (59.4%)	4 (50%)		30 (55.5%)	18 (67.8%)	
	Mixed	7 (9.5%)	1 (12.5%)		7 (13%)	1 (3.6%)	
Mitral Regurgitation Grade ^①^	Moderate to Severe	21 (28.4%)	2 (25%)	1	15 (27.8%)	8 (28.6%)	1
	Severe	53 (71.6%)	6 (75%)		39 (72.2%)	20 (71.4%)	
Mitral Valve EROA (cm^2^)		0.4 ± 0.21	0.64 ± 0.3	0.007	0.39 ± 0.18	0.5 ± 0.29	0.045
Mitral Valve Regurgitant Volume (mL)		56.4 ± 23.3	73.7 ± 35.1	0.086	55.3 ± 22.9	62.9 ± 28.3	0.227
Severe Tricuspid Regurgitation or Worse ^②^		9 (12.2%)	2 (25%)	0.291	8 (14.8%)	3 (10.7%)	0.741
Systolic Pulmonary Artery Pressure (mmHg)		49.4 ± 14.4	44.6 ± 15	0.411	48.2 ± 14.2	50.4 ± 15.1	0.533

Baseline echocardiography data are stratified according to a cut-off of rMR-2 at the completion of TEEMr and according to a cut-off of rMR-1 at discharge. Continuous variables are presented as the mean and standard deviation. Categorical variables are presented as percentages and absolute numbers. TEEMr, Transcatheter mitral valve repair; TEE, Transesophageal echocardiography; TTE, Transthoracic echocardiography; EROA, Effective regurgitant orifice area; ^①^ Mitral regurgitation grade was evaluated according to the American college of cardiology system of staging; ^②^ Tricuspid regurgitation staging was evaluated according to the American college of cardiology system of staging.

**Table 3:** (**a**) Univariable and multivariable Cox Proportional Hazards Regression Analysis for the occurrence all cause Death. (**b**) Univariable and multivariable Cox Proportional Hazards Regression Analysis for the occurrence all cause death or heart failure related hospitalization.

**(a)**						
	**Univariable Analysis**			**Multivariable Analysis**		
**Variable**	**HR**	**95% CI**	***p* Value**	**HR**	**95% CI**	***p* Value**
Diabetes Mellitus	3.18	1.76–5.73	<0.001	4.28	1.66–11.06	0.003
eGFR	0.97	0.95–0.99	0.004	0.95	0.92–0.98	<0.001
Past MI	2.67	0.82–8.7	0.104			
NYHA FC	2.03	0.99–3.98	0.111			
Age	1.02	0.97–1.08	0.382			
Gender	1.06	0.46–2.46	0.891			
Hypertension	2.04	0.47–8.78	0.336			
IHD	1.65	0.61–4.49	0.323			
Atrial fibrillation	0.84	0.35–1.97	0.695			
Hemoglobin	0.77	0.59–1.02	0.112			
Albumin	0.52	0.22–1.17	0.115			
Urgency of procedure	1.77	0.60–5.19	0.296			
ACS within 90 days	0.82	0.24–2.82	0.762			
STS score	1.02	0.99–1.07	0.147			
LVEDV	1	0.99–1.01	0.767			
LVESV	1	0.99–1.01	0.695			
LVEF	0.98	0.96–0.99	0.05	0.93	0.87–0.99	0.018
rMR ≤ 1 vs. rMR-2; TEEMr Completion	0.59	0.21–1.65	0.316			
rMR ≤ 2 vs. rMR > 2; TEEMr Completion	0.21	0.07–0.6	0.004	0.08	0.01–0.83	0.035
MVPG; TEEMr Completion	1.04	0.77–1.4	0.8			
rMR ≤ 1 vs. rMR-2; Discharge	0.49	0.18–1.34	0.165			
rMR ≤ 2 vs. rMR > 2; Discharge	0.95	0.28–3.24	0.937			
MVPG; Discharge	1.08	0.88–1.33	0.448			
**(b)**						
	**Univariable Analysis**			**Multivariable Analysis**		
**Variable**	**HR**	**95% CI**	***p* Value**	**HR**	**95% CI**	***p* Value**
Diabetes Mellitus	2.76	1.74–4.36	0.001	2.37	1.34–4.18	0.003
eGFR	0.98	0.97–1.00	0.122			
Past MI	2.42	1.16–5.05	0.019	2.39	0.77–7.08	0.133
NYHA	1.5	1.01–2.67	0.05	2.12	0.97–4.61	0.059
Age	0.98	0.94–1.02	0.355			
Gender	0.78	0.39–1.55	0.49			
Hypertension	1.34	0.51–3.46	0.545			
IHD	1.85	0.83–4.08	0.127			
Atrial fibrillation	0.54	0.27–1.10	0.091			
Hemoglobin	0.96	0.77–1.19	0.731			
Albumin	0.52	0.26–1.06	0.072			
Urgency of procedure	0.96	0.57–1.62	0.897			
ACS within 90 days	1.39	0.60–3.24	0.437			
STS score	1.0	0.96–1.04	0.913			
LVEDV	1.0	0.99–1.01	0.186			
LVESV	1.0	0.99–1.01	0.348			
LVEF	0.98	0.95–1	0.095			
rMR ≤ 1 vs. rMR-2; TEEMr Completion	0.68	0.41–2	0.804			
rMR ≤ 2 vs. rMR > 2; TEEMr Completion	0.35	0.12–1	0.06			
MVPG; TEEMr Completion	1.08	0.85–1.37	0.53			
rMR ≤ 1 vs. rMR-2; Discharge	0.48	0.2–0.98	0.045	0.24	0.08–0.73	0.012
rMR ≤ 2 vs. rMR > 2; Discharge	1.35	0.48–3.85	0.57			
MVPG; Discharge	1.09	0.93–1.28	0.272			

Univariable and multivariable Cox Proportional Hazards Regression Analyses. The tables presents the hazard ratios (HR) and 95% Confidence intervals (CI) for the occurrence all cause Death (Table 3(a)) and for the occurrence all cause Death or heart failure related hospitalization (Table 3(b)). eGFR, estimated glomerular filtration rate; MI, myocardial infarction; NYHA FC, New-York heart association functional class; IHD, ischemic heart disease; ACS, acute coronary syndrome; STS, society of thoracic surgeons; LVEDV, left ventricle end diastolic volume; LVESV, left ventricle end systolic volume; LVEF, left ventricle ejection fraction; rMR, residual mitral regurgitation; TEEMr, transcatheter edge-to-edge mitral valve repair; MVPG, mitral valve pressure gradient.

## Data Availability

The data presented in this study was obtained from PMC’s local TEEMr prospective registry and is available on request from the corresponding author.

## References

[1] Feldman T., Foster E., Glower D.D., Kar S., Rinaldi M.J., Fail P.S., Smalling R.W., Siegel R., Rose G.A., Engeron E. (2011). Percutaneous Repair or Surgery for Mitral Regurgitation. N. Engl. J. Med..

[2] Sorajja P., Vemulapalli S., Feldman T., Mack M., Holmes D.R., Stebbins A., Kar S., Thourani V., Ailawadi G. (2017). Outcomes ith Transcatheter Mitral Valve Repair in the United States: An STS/ACC TVT Registry Report. J. Am. Coll. Cardiol..

[3] Toggweiler S., Zuber M., Sürder D., Biaggi P., Gstrein C., Moccetti T., Pasotti E., Gaemperli O., Faletra F., Petrova-Slater I. (2014). Two-year outcomes after percutaneous mitral valve repair with the MitraClip system: Durability of the procedure and predictors of outcome. Open Heart.

[4] Kaneko H., Neuss M., Weissenborn J., Butter C. (2017). Impact of residual mitral regurgitation after MitraClip implantation. Int. J. Cardiol..

[5] Paranskaya L., D’Ancona G., Bozdag-Turan I., Akin I., Kische S., Turan G.R., Rehders T., Ortak J., Nienaber C.A., Ince H. (2013). Residual mitral valve regurgitation after percutaneous mitral valve repair with the MitraClip^®^ system is a risk factor for adverse one-year outcome. Catheter. Cardiovasc. Interv..

[6] Reichart D., Kalbacher D., Rübsamen N., Tigges E., Thomas C., Schirmer J., Reichenspurner H., Blankenberg S., Conradi L., Schäfer U. (2020). The impact of residual mitral regurgitation after MitraClip therapy in functional mitral regurgitation. Eur. J. Heart Fail..

[7] Neuss M., Schau T., Isotani A., Pilz M., Schöpp M., Butter C. (2017). Elevated Mitral Valve Pressure Gradient After MitraClip Implantation Deteriorates Long-Term Outcome in Patients With Severe Mitral Regurgitation and Severe Heart Failure. JACC Cardiovasc. Interv..

[8] Patzelt J., Zhang W., Sauter R., Mezger M., Nording H., Ulrich M., Becker A., Patzelt T., Rudolph V., Eitel I. (2019). Elevated Mitral Valve Pressure Gradient Is Predictive of Long-Term Outcome After Percutaneous Edge-to-Edge Mitral Valve Repair in Patients With Degenerative Mitral Regurgitation ( MR ), But Not in Functional MR. J. Am. Heart Assoc..

[9] Halaby R., Herrmann H.C., Gertz Z.M., Lim S., Kar S., Lindenfeld J., Abraham W.T., Grayburn P.A., Naidu S., Asch F.M. (2021). Effect of Mitral Valve Gradient After MitraClip on Outcomes in Secondary Mitral Regurgitation: Results From the COAPT Trial. JACC Cardiovasc. Interv..

[10] Öztürk C., Sprenger K., Tabata N., Sugiura A., Weber M., Nickenig G., Schueler R. (2021). The predictive value of intraprocedural mitral gradient for outcomes after MitraClip and its peri-interventional dynamics. Echocardiography.

[11] Orban M., Orban M., Lesevic H., Braun D., Deseive S., Sonne C., Hutterer L., Grebmer C., Khandoga A., Pache J. (2017). Predictors for long-term survival after transcatheter edge-to-edge mitral valve repair. J. Intervent. Cardiol..

[12] Lim D.S., Reynolds M.R., Feldman T., Kar S., Herrmann H.C., Wang A., Whitlow P.L., Gray W.A., Grayburn P., Mack M.J. (2014). Improved functional status and quality of life in prohibitive surgical risk patients with degenerative mitral regurgitation after transcatheter mitral valve repair. J. Am. Coll. Cardiol..

[13] Buzzatti N., De Bonis M., Denti P., Barili F., Schiavi D., Di Giannuario G., La Canna G., Alfieri O. (2016). What is a ‘good’ result after transcatheter mitral repair? Impact of 2+ residual mitral regurgitation. J. Thorac. Cardiovasc. Surg..

[14] Puls M., Tichelbäcker T., Bleckmann A., Hünlich M., Ehe K., von der Beuthner B.E., Rüter K., Beißbarth T., Seipelt R., Schöndube F. (2014). Failure of acute procedural success predicts adverse outcome after percutaneous edge-to-edge mitral valve repair with MitraClip. EuroIntervention J. Eur. Collab. Work Group Interv. Cardiol. Eur. Soc. Cardiol..

[15] Capodanno D., Adamo M., Barbanti M., Giannini C., Laudisa M.L., Cannata S., Curello S., Immè S., Maffeo D., Bedogni F. (2015). GRASP-IT Investigators. Predictors of clinical outcomes after edge-to-edge percutaneous mitral valve repair. Am. Heart J..

[16] Sürder D., Pedrazzini G., Gaemperli O., Biaggi P., Felix C., Rufibach K., Maur CA der Jeger R., Buser P., Kaufmann B.A., Moccetti M. (2013). Predictors for efficacy of percutaneous mitral valve repair using the MitraClip system: The results of the MitraSwiss registry. Heart Br. Card. Soc..

[17] Rudski L.G., Lai W.W., Afilalo J., Hua L., Handschumacher M.D., Chandrasekaran K., Solomon S.D., Louie E.K., Schiller N.B. (2010). Guidelines for the echocardiographic assessment of the right heart in adults: A report from the American Society of Echocardiography endorsed by the European Association of Echocardiography, a registered branch of the European Society of Cardiology, and the Canadian Society of Echocardiography. J. Am. Soc. Echocardiogr..

[18] Zoghbi W.A., Asch F.M., Bruce C., Gillam L.D., Grayburn P.A., Hahn R.T., Inglessis I., Islam A.M., Lerakis S., Little S.H. (2019). Guidelines for the Evaluation of Valvular Regurgitation After Percutaneous Valve Repair or Replacement: A Report from the American Society of Echocardiography Developed in Collaboration with the Society for Cardiovascular Angiography and Interventions, Japanese Society of Echocardiography, and Society for Cardiovascular Magnetic Resonance. J. Am. Soc. Echocardiogr..

[19] Foster E., Wasserman H.S., Gray W., Homma S., Di Tullio M.R., Rodriguez L., Stewart W.J., Whitlow P., Block P., Martin R. (2007). Quantitative assessment of severity of mitral regurgitation by serial echocardiography in a multicenter clinical trial of percutaneous mitral valve repair. Am. J. Cardiol..

[20] Stone G.W., Adams D.H., Abraham W.T., Kappetein A.P., Généreux P., Vranckx P., Mehran R., Kuck K.-H., Leon M.B., Piazza N. (2015). Mitral Valve Academic Research Consortium (MVARC). Clinical Trial Design Principles and Endpoint Definitions for Transcatheter Mitral Valve Repair and Replacement: Part 2: Endpoint Definitions: A Consensus Document From the Mitral Valve Academic Research Consortium. J. Am. Coll. Cardiol..

[21] Tabata N., Weber M., Sugiura A., Öztürk C., Ishii M., Tsujita K., Nickenig G., Sinning J.-M. (2019). Impact of the Leaflet-to-Annulus Index on Residual Mitral Regurgitation in Patients Undergoing Edge-to-Edge Mitral Repair. JACC Cardiovasc. Interv..

[22] Herrmann H.C., Rohatgi S., Wasserman H.S., Block P., Gray W., Hamilton A., Zunamon A., Homma S., Di Tullio M.R., Kraybill K. (2006). Mitral valve hemodynamic effects of percutaneous edge-to-edge repair with the MitraClip device for mitral regurgitation. Catheter Cardiovasc. Interv..

[23] Boerlage-van Dijk K., van Riel A.C.M.J., de Bruin-Bon R.H.A.C.M., Wiegerinck E.M.A., Koch K.T., Vis M.M., Meregalli P.G., Bindraban N.R., Mulder B.J.M., Piek J.J. (2014). Mitral inflow patterns after MitraClip implantation at rest and during exercise. J. Am. Soc. Echocardiogr..

[24] Biaggi P., Felix C., Gruner C., Herzog B.A., Hohlfeld S., Gaemperli O., Stähli B.E., Paul M., Held L., Tanner F.C. (2013). Assessment of mitral valve area during percutaneous mitral valve repair using the MitraClip system: Comparison of different echocardiographic methods. Circ. Cardiovasc. Imaging.

[25] Herrmann H.C., Kar S., Siegel R., Fail P., Loghin C., Lim S., Hahn R., Rogers J.H., Bommer W.J., Wang A. (2009). Effect of percutaneous mitral repair with the MitraClip device on mitral valve area and gradient. EuroIntervention J. Eur. Collab. Work. Group Interv. Cardiol. Eur. Soc. Cardiol..

[26] Grayburn P.A., She L., Roberts B.J., Golba K.S., Mokrzycki K., Drozdz J., Cherniavsky A., Przybylski R., Wrobel K., Asch F.M. (2015). Comparison of Transesophageal and Transthoracic Echocardiographic Measurements of Mechanism and Severity of Mitral Regurgitation in Ischemic Cardiomyopathy (from the Surgical Treatment of Ischemic Heart Failure Trial). Am. J. Cardiol..

[27] Honjo O., Kotani Y., Osaki S., Fujita Y., Suezawa T., Tateishi A., Ishino K., Kawada M., Akagi T., Sano S. (2006). Discrepancy between intraoperative transesophageal echocardiography and postoperative transthoracic echocardiography in assessing congenital valve surgery. Ann. Thorac. Surg..

[28] Saiki Y., Kasegawa H., Kawase M., Osada H., Ootaki E. (1998). Intraoperative TEE during mitral valve repair: Does it predict early and late postoperative mitral valve dysfunction?. Ann. Thorac. Surg..

[29] Dietl A., Prieschenk C., Eckert F., Birner C., Luchner A., Maier L.S., Buchner S. (2018). 3D vena contracta area after MitraClip© procedure: Precise quantification of residual mitral regurgitation and identification of prognostic information. Cardiovasc. Ultrasound.

[30] Patzelt J., Zhang Y., Seizer P., Magunia H., Henning A., Riemlova V., Patzelt T.A.E., Hansen M., Haap M., Riessen R. (2016). Effects of Mechanical Ventilation on Heart Geometry and Mitral Valve Leaflet Coaptation During Percutaneous Edge-to-Edge Mitral Valve Repair. JACC Cardiovasc. Interv..

[31] Rahimtoola S.H., Durairaj A., Mehra A., Nuno I. (2002). Current Evaluation and Management of Patients With Mitral Stenosis; American Heart Association. Circulation.

[32] Baumgartner H., Hung J., Bermejo J., Chambers J.B., Evangelista A., Griffin B.P., Iung B., Otto C.M., Pellikka P.A., Quiñones M. (2009). American Society of Echocardiography, European Association of Echocardiography. Echocardiographic assessment of valve stenosis: EAE/ASE recommendations for clinical practice. J. Am. Soc. Echocardiogr..

[33] Lin B.A., Forouhar A.S., Pahlevan N.M., Anastassiou C.A., Grayburn P.A., Thomas J.D., Gharib M. (2010). Color Doppler jet area overestimates regurgitant volume when multiple jets are present. J. Am. Soc. Echocardiogr..

[34] Voelker W., Regele B., Dittmann H., Mauser M., Ickrath O., Schmid K.M., Karsch K.R. (1992). Effect of heart rate on transmitral flow velocity profile and Doppler measurements of mitral valve area in patients with mitral stenosis. Eur. Heart. J..

[35] Gorlin R., Gorlin S.G. (1951). Hydraulic formula for calculation of the area of the stenotic mitral valve, other cardiac valves, and central circulatory shunts. I. Am. Heart J..

[36] Hoffmann R., Altiok E., Reith S., Brehmer K., Almalla M. (2014). Functional effect of new atrial septal defect after percutaneous mitral valve repair using the MitraClip device. Am. J. Cardiol..

[37] Smith T., McGinty P., Bommer W., Low R.I., Lim S., Fail P., Rogers J.H. (2012). Prevalence and echocardiographic features of iatrogenic atrial septal defect after catheter-based mitral valve repair with the MitraClip system. Catheter. Cardiovasc. Interv. Off. J. Soc. Card. Angiogr. Interv..

